# A rare primary posterior mediastinal angiolipoma: A case report

**DOI:** 10.1016/j.rmcr.2021.101536

**Published:** 2021-10-21

**Authors:** Noni Novisari Soeroso, Fannie Rizki Ananda, Maulidya Ayudika Dandanah

**Affiliations:** a Thoracic Oncology Division, Department of Pulmonology and Respiratory Medicine, Faculty of Medicine, Universitas Sumatera Utara, Indonesia; b Department of Pulmonology and Respiratory Medicine, Faculty of Medicine, Universitas Sumatera Utara, Indonesia; c Thoracic Surgery Division, Department of Surgery, Faculty of Medicine, Universitas Sumatera Utara, Indonesia

**Keywords:** Mediastinal tumor, Angiolipoma, Exploration thoracotomy

## Abstract

*Mediastinal angiolipoma* is a rare benign neoplasm composed of mature adipose tissue with an abnormal vessel. Owing to its unspecific symptoms and slow progressions, the diagnosis of this disease is often delayed by clinicians. Here we present a 68-years-old-woman presented with mild chest pain radiates to the back with chronic dry cough. Chest imaging showed right medial posterior intrathoracic mass attached to 3rd-5th vertebrae body without bone destructions. Exploratory thoracotomy with segmentectomy was successfully performed. Histopathology examinations showed adipose tissue surrounded by a blood vessel nest, typical for angiolipoma. The patient showed rapid recovery and was discharged a week after the surgery. After two years of follow-up, the patient showed no sign of tumor recurrence and was clinically stable. This case is the first occurrence of non-infiltrating mediastinal angiolipoma reported in Southeast Asia.

## Highlight

• This is the first case report of non-infiltrating mediastinal angiolipoma in Southeast Asia.
• Histopathology examination may be performed by using an invasive or non-invasive procedure.
• In developing countries where VATS cannot be carried out, early complete resection is essential to get favorable outcomes.

## Abbreviations

AFP: Alpha-fetoprotein
β-HCG: Beta-human chorionic gonadotropin
CEA: Carcinoembryonic antigen
CECT: Contrast-Enhanced Computed Tomography
HE: Hematoxylin-Eosin
LDH: Lactate Dehidrogenase
USG: Ultrasonography
VAS: Visual Analog Scale
VATS: Video-Assisted Thoracoscopic Surgery

## 1 Background

*Mediastinal angiolipoma* is an extremely rare disease composed of mature adipocytes mixed with vascular components [1,2]. The location of this tumor was mainly in the limbs [3] and the trunk, making the mediastinal findings of this tumor often misdiagnosed with other types of posterior mediastinal tumor [4]. According to the literature, there were two entities in angiolipoma; infiltrating and noninfiltrating tumors [3].

Here we present a case report of a 68-years-old woman diagnosed with posterior mediastinal angiolipoma without infiltrations to the spinal nerve and successfully resected in open thoracotomy.

## 2 Case reports

**Fig. 1:**
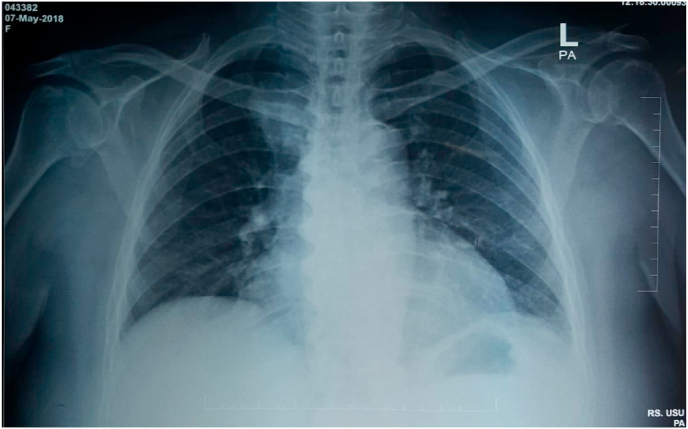
Plain Chest X-Ray of 68 years old woman with mediastinal angiolipoma. Plain Chest X-Ray showed a right paratracheal mass (arrows) with a well-demarcated lesion attached to the mediastinal cage.

**Fig. 2:**
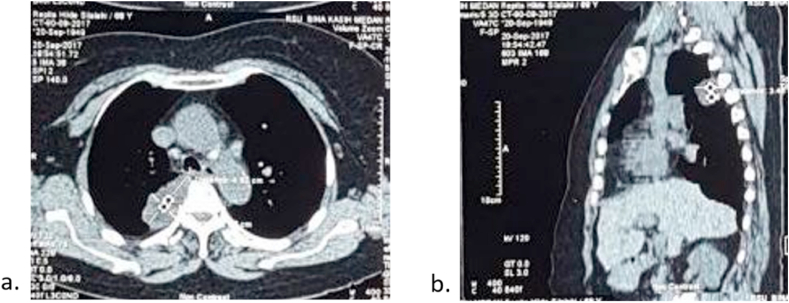
Contrast-enhanced Computed Tomography. (a) axial view (b) sagittal view. Figures (a) and (b) showed a lobulated lesion in the right medial posterior intrathoracic with an attachment to the right 3rd-5th vertebral body and mild right pleural effusion.

**Fig. 3:**
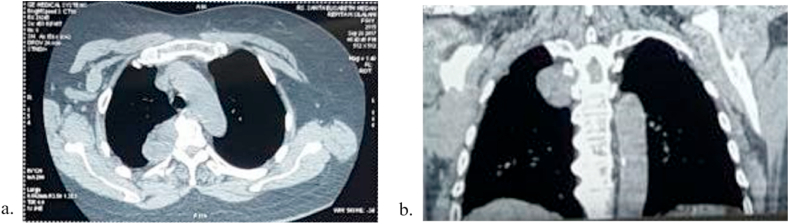
Follow-up contrast-enhanced CT in 12th January 2018 (a) axial view (b) coronal view. Figures (a) and (b) showed a lobulated mass in the right medial posterior intrathoracic mass attached to the 3rd-5th vertebrae body with size 5x3.2 × 2.8cm and without connection to the spinal canal. There is no destruction of vertebrae bone. Compared with recent Chest CT, there was no significant gain of mass.

A 68-years-old woman was brought to the hospital due to chest pain that radiated to her back for the last six months. The characteristics of pain were like stabbing with low intensity (VAS 3). Dry cough was experienced intermittently in the last six months. However, no other respiratory symptoms were presented. She was a secondhand smoker with no history of biomass exposure. The vital sign was remained normal, with respiratory examinations showed diminished breath sound in the right perihilar. Plain Chest X-Ray revealed a regular mass in the right paratracheal (Fig. 1). Contrast-Enhanced Computed Tomography (CECT) of the chest showed a lobulated mass in posterior media mediastinum attached to right 3rd-5th thoracal rib with pleural effusion and cardiomegaly (Fig. 2a and b). Follow-up contrast-enhanced CT three months later presented hypodense lobulated mass, measuring 5 x 3.2 × 2.8cm in right posterior medial mediastinum attached to 3rd-5th rib without any attachment to the spinal nerve and any destruction in spinal bone. Compared with the previous CECT, there was a minimally progressed tumor (Fig. 3a and b). Laboratory findings and the tumour markers, including AFP, β-HCG, LDH, and CEA, remained normal.

**Fig. 4:**
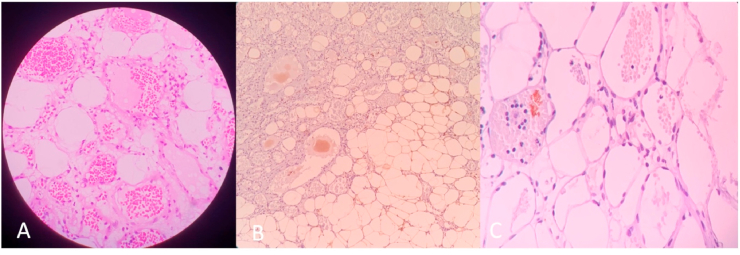
(A) Angiolipoma features of HE staining showed mature adipose tissue surrounded by blood vessels nest (B) Immunohistochemistry S100 showed positive of adipose cells (C) Ki67 index revealed low proliferation typical for a benign lesion.

After delayed operation due to patient preference, exploration thoracotomy with segmentectomy was performed under general anesthesia. From the intraoperative procedure, giant tumor with size 8x6x3cm adhered to right 3rd costae and 4th thoracal vertebrae was removed. Macroscopic appearance showed giant, solid, and springy tumor. There was no complication during surgery and the posterior mediastinal mass was successfully removed. Histopathology preparation revealed mature adipose tissue with multiple small blood vessels with Hematoxylin-Eosin (HE) staining (Fig. 4A). Further examination with Immunohistochemistry S100 showed positive adipose cells (Fig. 4B) and the Ki67 index revealed low proliferation, typical for benign lesions (Fig. 4C).

**Fig. 5:**
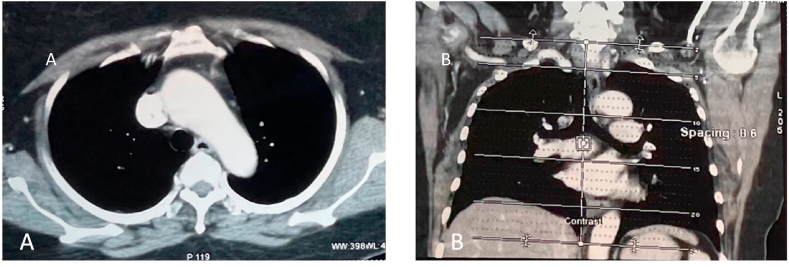
Follow up contrast-enhanced CT in 20^th^ February 2020 (a) axial view (b) coronal view. Figures (a) and (b) showed normal appearance of the mediastinal window with no sign of tumor occurrence or any other abnormalities.

There were no complications after surgery and the patient was discharged 7 days after surgery without any significant clinical symptoms. There was no radiotherapy adjuvant because the mass was resected without any residual. Two years of follow-up was undergoing, and she remained fit with no signs of tumour recurrence. Followed up CECT 3 years later confirmed the absence of tumour recurrence (Fig. 5).

## 3 Discussions

Angiolipoma is a rare disease origin from mesenchymal tissue. It is defined as benign mature adipose tissue and blood vessel, more common in extremity subcutaneous tissue and muscles [[1], [2], [3], [4]]. Lin et al. described the characteristics of the tumor as tumor formation may be encapsulated or without a capsule, composed >50% of mature adipose tissue, blood proliferation seen microscopically [3]. The histology classification was divided into two categories, including infiltrating and non-infiltrating angiolipoma [3]. This case showed infiltrating angiolipoma with blood vessel nesting in HE staining. Infiltrating angiolipoma is characterized by non-capsulated tumors and attached to adjacent tissue. Meanwhile, non-infiltrating angiolipoma is characterized by the encapsulated tumor, which could suppress the adjacent tissue and give manifestation such as pain [5]. Further, immunohistochemistry examination was needed to differentiate angiolipoma from other benign tumors. Melan A, Smooth muscle actin, S-100, cytokeratin, Ki67 were common antibodies used in confirming the diagnosis of angiolipoma [6]. However, there was no definite IHC antibody used to confirm the diagnosis of angiolipoma because histopathology appearance was sufficient for diagnosing angiolipoma. Fibrin thrombi within vessels is a hallmark of angiolipoma in histopathology preparation with HE staining [7]. In this case, we carried out S100 and Ki67 antibodies as additional follow-up for diagnosing angiolipoma. S100 was used to confirm the adipocyte cells [8] compound in tumors while Ki67 described the proliferation degree in tumor cells of which represents the malignancy tendency [9].

Compression and invasion to the adjacent tissue presented unspecific clinical manifestations. The patient might experience chest pain, cough, dyspnea, and developed obstructive pneumonitis [4]. Furthermore, neuronal manifestation resulting from spinal compression might result in back pain, lower extremity weakness, and numbness [1,10]. In this case, the patient's main complaint was mild chest pain and chronic cough resulting from compression to the main airway and mediastinum cage. There was no neuronal manifestation owing to the absence of vertebrae destruction and spinal involvement.

Moreover, chest pain might be associated with the right pleural effusion that often occurs in mediastinal angiolipoma [5,11,12]. Pleural effusion might occur due to tumor invasion in pleural space or mesothelium reacting to an unknown stimulus [12]. The findings of cell tumors in pleural fluid analysis can execute the etiology of pleural effusions. In this case, a pleural puncture with USG guiding was carried out, but no pleural fluid was aspirated due to a small amount of pleural fluid.

Optimal management of angiolipoma is still being argued due to the low number of cases. However, conservative management with follow-up can be considered in the case of asymptomatic and small-sized tumors. It should be noted that there was no definite guideline in assessing the definition of small-sized tumors [12]. Surgical excision and or transarterial embolization should be performed in symptomatic or large tumor cases [1,2,4,5]. In incomplete resection due to large tumor size or the involvement of vital organs, postoperative radiotherapy should be considered to minimalize the recurrence rate [3]. In this case, in early presentation, the patient refused to get surgery, but after few months of conservative management, she agreed to undergo thoracotomy with segmentectomy. The tumor resection was complete, so there was no need for adjuvant radiotherapy. The recurrence of angiolipoma was high, and 1–2 years follow-up should be arranged for preventing complications due to invasion to surrounding tissue [[2], [3], [4]]. In this case, we have evaluated the patient for three years and showed no sign of tumor recurrence or any residual tumor confirmed by CECT.

## 4 Conclusions

Mediastinal angiolipoma is an extremely rare benign tumor where unspecific clinical manifestations often delay the diagnosis. Therefore, early management strategies were needed to prevent invasions to adjacent tissue and lead to difficult resection. This tumor has a high recovery rate if treated well, so the clinicians and surgeons must be aware of this type of mediastinal tumor.

## Funding

This study did not receive any external funding from commercial or non-profit sectors in collecting, interpreting data, writing, and publishing the manuscript.

## Ethical considerations

All procedures have been done according to the Declaration of Helsinki, and written informed consent was obtained from the subjects involved in the study, including the picture. Ethical approval is not applicable since it is a case report.

## Data availability statement

The generated and analyzed datasets during the current study are available upon reasonable request to the corresponding authors.

## Declaration of competing interest

The authors have no financial or personal circumstances with pharmacists or organizations that could influence the originality of this manuscript.

## CRediT authorship contribution statement

**Noni Novisari Soeroso:** Conceptualization, Data curation, Formal analysis, Investigation, Methodology, Project administration, Resources, Supervision, Validation, Visualization, Writing – original draft, Writing – review & editing. **Fannie Rizki Ananda:** Conceptualization, Data curation, Formal analysis, Writing – original draft, Writing – review & editing. **Maulidya Ayudika Dandanah:** Conceptualization, Investigation, Methodology, Resources, Supervision, Validation, Visualization, Writing – review & editing.
